# Stability of Glutaraldehyde in Biocide Compositions

**DOI:** 10.3390/ijms21093372

**Published:** 2020-05-10

**Authors:** Alina Matei, Cristina Puscas, Iulia Patrascu, Maria Lehene, Julia Ziebro, Florina Scurtu, Monica Baia, Dan Porumb, Robert Totos, Radu Silaghi-Dumitrescu

**Affiliations:** 1 Department of Chemistry, Babes-Bolyai University, 1 Mihail Kogalniceanu street, 400084 Cluj-Napoca, Romania; mateialina@chem.ubbcluj.ro (A.M.); cbischin@chem.ubbcluj.ro (C.P.); iuliapatrascu9416@yahoo.com (I.P.); mlehene@chem.ubbcluj.ro (M.L.); florinadeac@chem.ubbcluj.ro (F.S.); idporumb@gmail.com (D.P.); rtotos@chem.ubbcluj.ro (R.T.); 2 Department of Chemistry, Northern Kentucky University, Southgate, KY 41099, USA; ziebroj1@mymail.nku.edu; 3 Department of Physics, Babes-Bolyai University, 1 Mihail Kogalniceanu street, 400084 Cluj-Napoca, Romania; monica.baia@phys.ubbcluj.ro

**Keywords:** glutaraldehyde, biocide, SDS-PAGE, DFT, Raman, NMR, LC–MS, titration

## Abstract

Glutaraldehyde (GA) is used as biocide in hospitals. Recent public investigations on the chemical composition of biocides used in Romania have in some cases found GA, as a key ingredient, to be apparently diluted. However, these data did not explicitly consider the complex chemical equilibria inherent to GA. An investigation of experimental and theoretical data is reported here, assessing the stability of GA solutions relevant for biocide compositions. GA solutions of various chemical composition and under varying circumstances were analyzed using spectroscopy (UV-VIS, Raman, NMR) coupled with density functional theory (DFT) calculations, as well as chemically, such as via the formation of imines in reaction/titration with glycine monitored at 270 nm; using LC-MS; or using SDS-PAGE analysis with GA as reagent in the polymerization of two test proteins- hemoglobin and myoglobin. The spectral properties of GA changed significantly over time, in a temperature-dependent manner; titration with glycine confirmed the spectral data. SDS-PAGE experiments demonstrated a non-linear and apparently unpredictable change in the reactivity of GA over time. The results may be relevant for the determination of GA concentration in various settings such as biocide analysis, hospital wastewaters, and others.

## 1. Introduction

Among the wide range of uses of glutaraldehyde (industrial, agricultural, medical), its application as biocide in hospital settings is among the most important [1]. Determination of glutaraldehyde (GA) concentration is essential in contexts such as conformity checks in hospital-used biocides [2] or toxicity assays [3] (GA is a volatile substance and the vapors can irritate the skin, eyes, nose, and lungs) [4]. 

**Scheme 1:**
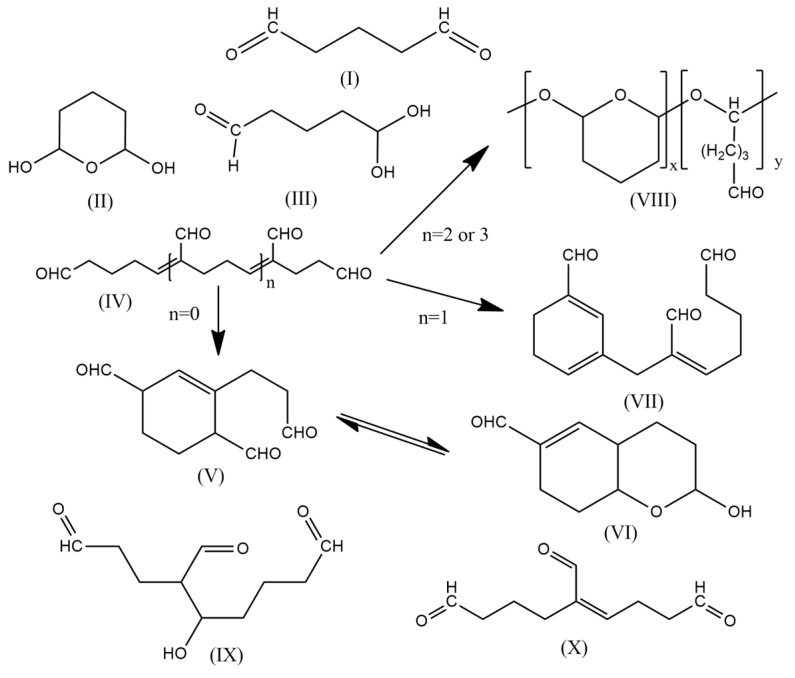
Chemical structures of products shown to be present in glutaraldehyde (GA) solutions.

Commercial GA is typically available at a 25% nominal concentration, but its concentration in biocides may vary. There are a few methods for the determination of GA concentrations. One option involves spectrophotometric titration using a mixture of phenol solution and perchloric acid [4]. This method is used only for methanolic samples. Other methods involve column chromatography [5,6,7,8], electrophoresis [9], or iodometric titration [10]. However, even in “pure” solutions, GA (I in Scheme 1) undergoes a wide range of reactions, including at a first stage cyclization (II), hydrate formation (III), and dimerization via aldolic (IX) and crotonic condensation (X). Subsequent reactions also occur, as previously described. Scheme 1 illustrates the chemical structures of products previously shown to be present in GA solutions [11]. 

GA’s two aldehyde groups allow it to be used as a cross-linking reagent, targeting in particular the amino groups of proteins, producing polymeric as well as nonpolymeric species; at large concentrations, this crosslinking leads to biocidal effects [12]. Recent public investigations on the conformity of the chemical composition of biocides currently in use in Romanian hospitals [13,14] have reportedly found GA (as a key ingredient) to be up to 10-fold diluted with respect to the nominal concentration listed for the respective products. However, these initial chemical analyses were only measuring the concentration of monomeric GA, in contrast to a wealth of data (cf. Scheme 1), according to which “pure” GA solutions, in fact, contain a long list of dimers, oligomers, and condensation products, among others [15]. These condensation/oligomerisation products are difficult to contain and quantify under normal conditions. Nevertheless, they often still preserve functional aldehyde groups, which allows them to still be efficient biocides. Hence, the concentration of monomeric GA appears to be hardly relevant when considering the conformity of a biocide; nevertheless, this obvious conclusion is occasionally ignored, sometimes with absolutely dramatic implications on a nation-wide scale [13,14]. 

Reported here is an exploration of experimental and theoretical data that may allow more informed analyses of GA concentrations in biocides as well as generally in GA-containing solutions. Considered here are GA solutions of various chemical compositions and under varying storage conditions: room temperature, under light or dark, or at 40 °C for several weeks. These circumstances were chosen in order to assess/evaluate the possibility of decreases in the efficiency of biocides under various conditions of storage known to occur de facto from production facilities to hospitals. Several lines of analysis were employed: UV-VIS spectroscopy, Raman spectroscopy, nuclear magnetic resonance spectroscopy (NMR), titrations, SDS-PAGE analysis, liquid chromatography coupled with mass spectrometry (LC-MS), and density functional theory (DFT) calculations. The results may be taken as relevant for the determination of GA concentration in various settings (biocide analysis, hospital wastewaters, and others).

## 2. Results and Discussion

### 2.1. Raman Spectra

**Figure 1:**
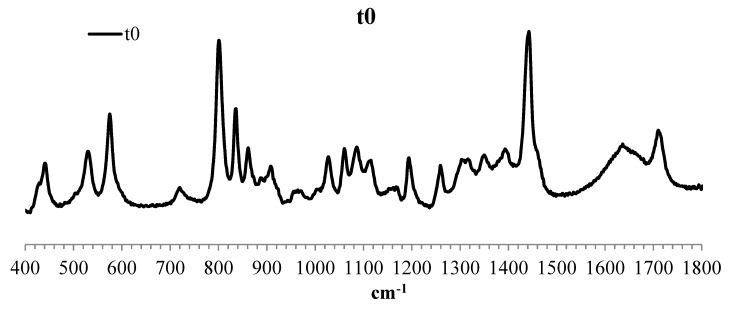
Raman spectra of 1M GA incubated at the initial time (t0), room temperature, and 40 °C for 8 weeks.

Figure 1 shows the Raman changes for a GA solution after incubation at room temperature or at 40 °C for 8 weeks (see also Figures S1 and S2 for concentration dependencies). The relative ratio of the peaks at 1440 (CH_(2)_ vibrations) vs. 1710 cm^−1^ (carbonyl vibrations) decreased by 15% in the room temperature sample and by 36% in the 40 °C sample. This decrease in the contributions of the CH moieties vs. the carbonyl moieties can be interpreted as evidence for degradation of the GA, as previously described [11], to multiple products formed via aldol-type condensation where carbonyl groups are still present; the diversity of the hydrocarbon skeletons of these products would imply that they would not be easily detected as individual bands (see, for example, the DFT-computed spectra for a series of simple GA derivatives in Figure S3, and the diversity of structures in Scheme 1), but rather indirectly via the decrease in the contribution of the CH_(2)_ groups to the spectrum, whereas the remaining carbonyl groups, regardless of the skeleton to which they are attached, would still be detected at the same wavelength. If one considers the computed spectra for GA derivatives in Figure S3, where some of the derivatives had partial overlap with the 1440 cm^−1^ band of GA, then the experimentally-observed changes of 15%–36% in the ratio of 1440 (CH_(2)_ vibrations) vs. 1710 cm^−1^ (carbonyl vibrations) in Figure 1 may well imply that after 8 weeks of storage the majority of the GA had been transformed into other compounds. In the 40 °C sample, one may also note a clear development of a band at 1640 cm^−1^, which, according to the DFT calculations, arose from carbon–carbon double bonds, which implies the presence of sizeable amounts of crotonic products and/or other unsaturated compounds. 

### 2.2. NMR Spectra

**Figure 2:**
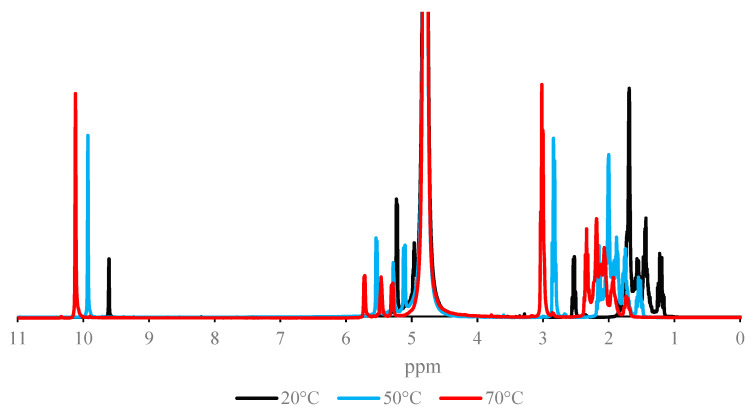
^1^H-NMR spectra of 12.5% glutaraldehyde in D_2_O at varying temperatures. Chemical shifts (parentheses show, in this order: the coupling type, coupling constant when not singlet, the integrated value, and the chemical assignment to the extent available/possible) in parts per million (ppm) for room temperature: 9.611 (singlet, 2H, CHO), 5.235 (doublet of doublets, 6 Hz/2Hz, 8.31H, unsaturated), 4.972 (sextet, 2.8 Hz, 6H, unsaturated), 2.533 (quartet of doublets, 7.2 Hz/1.2 Hz, 4.29H, aliphatic), 1.832 (multiplet, not determined (n.d.), 2.21H, aliphatic), 1.702 (multiplet, n.d., 27H aliphatic), 1.566 (multiplet, n.d., 12H, aliphatic), 1.445 (multiplet, n.d., 14H, aliphatic), 1.224 (multiplet, n.d., 7H, aliphatic). At 50 °C: 9.932 (singlet, 2H, CHO), 5.555 (doublet of doublets, 4.4 Hz/1.2Hz, 1.84H, unsaturated), 5.252 (multiplet, n.d., 1.34H, unsaturated), 5.152 (doublet of doublets, 9.6 Hz/2Hz, 1.73H, unsaturated), 2.825 (quartet, 6.8 Hz, 4.34H, aliphatic), 1.832 (multiplet, n.d., 1.91H, aliphatic), 1.224 (multiplet, n.d., 16H, aliphatic). At 70 °C: 10.120 (singlet, 2H, CHO), 5.723 (doublet, 4 Hz, 0.73H, unsaturated), 5.465 (multiplet, n.d., 0.67H, unsaturated), 5.300 (doublet, 10.4 Hz, 0.66H, unsaturated), 3.022 (quartet, 7.2 Hz, 4.14H, aliphatic), 2.338 (quintet, 8.4 Hz, 2.02H, aliphatic), 2.127 (multiplet, n.d., 7.35H, aliphatic), 1.933 (multiplet, n.d., 0.73H, aliphatic).

To further explore the composition and variability of glutaraldehyde solutions, NMR spectra were recorded for GA (25%) at 20, 50, and 70 °C after mixing with D_2_O (1:1 volume ratio). The spectra (Figure 2) showed signals in the aldehyde region (9.5–10 ppm), the aliphatic region (1–3 ppm), and in the region specific to protons directly attached to unsaturated groups (5–6 ppm). At any given temperature, only one signal was observed in the 9.5–10 ppm region; hence, either the aldehyde proton was identical between glutaraldehyde and all of its aldehyde-containing byproducts, or glutaraldehyde was the only aldehyde present. The 5–6 ppm signals were neither purely aliphatic nor aldehydic, and may hence be assigned to crotonic-type condensation products of aldehyde, but not to glutaraldehyde itself. The 1–3 ppm signals are expected to arise from glutaraldehyde as well as from its reaction/degradation products.

Assuming the 10 ppm signal to be solely due to intact glutaraldehyde (hence, an upper estimate of the GA concentration), and taking into account the stoichiometry within this compound (i.e., the sum of all integrals pertaining to GA must be four times the integral of the 10 ppm signal, as GA contains two aldehyde protons and a total of eight protons), ≈10% of the integrals in the room-temperature spectrum could be assigned to intact glutaraldehyde. This estimate was increased to 27% at 50 °C and to 43% at 70 °C. Notably, when the 70 °C sample was cooled back to room temperature, the (upper limit of) glutaraldehyde proportion was 13%, almost (but not completely) identical to the pre-heating stage. These data are in line with the Raman data discussed above, both in terms of pointing to glutaraldehyde being present as a mixture of several products and in terms of suggesting that thermal treatment/incubation may affect the chemical composition.

The data discussed above suggest rapid exothermic equilibria in which GL undergoes significant concentration changes with temperature. Relevantly, at various concentrations of GA (25%, 2.5%, and 0.25%) the relative intensities of the signals (aldehyde vs 5 ppm vs aliphatic) were not affected across these concentrations (data not shown). This suggests that GA dimerization (such as in an aldolic or crotonic condensation) was not a key component of the equilibria detectable in NMR.

Incubation of a ≈0.3 mM sample of GA for 1 week at room temperature led to no changes in the relative intensities of the signals, but did result in a decrease of their intensities (cf. Figure S4). This suggests a larger number of products, at low concentrations, as opposed to a small number of distinct products that would have been observable in NMR directly. The fast nature of the equilibria seen in Figure 1 suggests that simple condensation and/or hydration of GA is not the dominant reason why GA disappears at longer incubation time; polymerization of these products and/or their oxidation is instead proposed to be responsible for the changes seen after incubation of a longer time.

To verify the assignments discussed above, the NMR spectra for the glutaraldehyde and other products were computed using density functional theory. These data, shown in Table S1, confirm that the aldehyde protons in several products can indeed have very similar shieldings. In addition to this, these data suggest that the 5–6 ppm signals may have originated not only from crotonaldehyde but also from at least two other products (II and III in Scheme 1, the internal condensation product or the GA hydrate), but not from the aldolic dimer (IX). In fact, considering the lack of dependence on GA concentration noted above, one may assume that II or III were responsible for these signals.

The temperature variations of the NMR spectra offer an opportunity to derive thermodynamic parameters (cf. Table 1) on the basis of the estimates of GA concentrations discussed above. The increase of GA concentration with temperature, pointed out above, expectedly correlated with negative (though very small) ΔG values. Derived also from these data was a negative enthalpy change counterbalanced by a negative entropy change, thus leading to a near-zero free energy change. The very small negative entropy suggests that a dominant factor in the reaction was a condensation and/or an aldehyde hydration process (the energetics of which were in the same range on the basis of previous experimental data [16,17]). 

### 2.3. LC-MS Analysis

When subjected to LC-MS analysis, all analyzed samples presented two peaks in the chromatogram, at relatively conserved retention times and with relatively conserved intensities (cf. Table 2). The first chromatographic peak generally allowed detection of stronger MS signals (one order of magnitude compared to the second peak) that could be assigned to intact GA and to some of its reaction products (illustrated in Figure 1). The second peak, at least at lower GA concentrations, contained less or no detectable GA contribution, and instead featured several signals whose masses could not be straightforwardly connected to any of the structures illustrated in Scheme 1. 

Incubation of GA at 0.3 mM, representative of a concentration found in biocides, for 4 weeks at 40 °C, representative of improper storage conditions, led to an almost complete disappearance of the first LC peak—the one that in the respective control sample featured GA MS signals. This is in line with the Raman interpretations given above, suggesting that at this time point there was essentially no intact GA left in the sample. In a separate experiment, a more concentrated sample was aged for a shorter time but at a larger temperature (5 h at 70 °C). Here, the differences were smaller but did include slight changes in the MS profile of the second peak. 

### 2.4. Titration with Glycine

**Figure 3:**
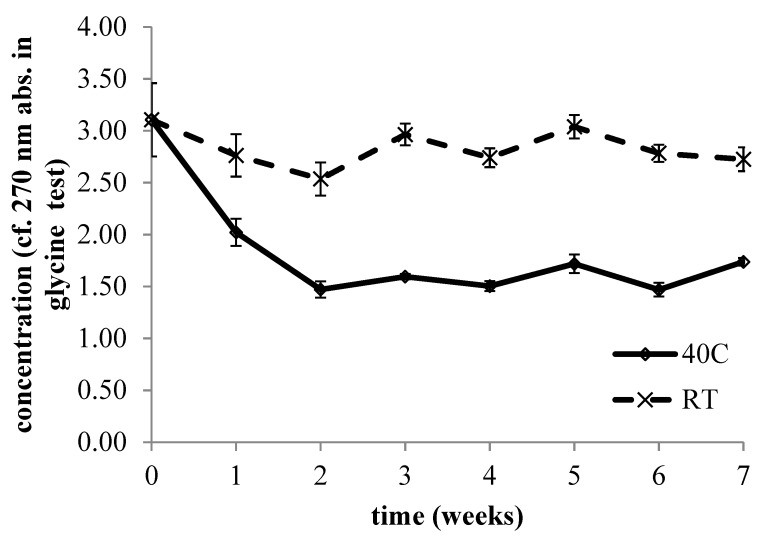
Time evolution for 8 weeks of GA concentration at 40 °C and at room temperature, as measured by titration with glycine.

Glutaraldehyde generally exerts its biocide activity by crosslinking the amine groups on proteins via imine group formation. Titration with amines has indeed been previously proposed as a tool for quantitating aldehydes in solution, following the distinct absorbance of the newly formed imine group [18]. Indeed, the imine reaction product resulting from glycine and GA can be followed at 270 nm, without interference from the much weaker absorption peaks of GA at 235 nm and 280 nm [19]. Figure 3 shows such data; after only 2 weeks of incubation at 40 °C, the concentration of glycine-titratable aldehyde groups (GTAP) appeared to be reduced to half. Considering that a simple aldol condensation reduced the number of titratable groups by one third, Figure 2 then suggests that all of the GA had condensed already after 2 weeks at 40 °C. Moreover, the missing 50% of non-GTAP aldehyde groups were not transformed into any form that would revert to GTAP via equilibria on the time scale of the experiment (minutes to hours). Smaller variations of GTAP were also seen in the sample incubated at room temperature.

### 2.5. Efficiency on Protein Crosslinking (SDS-PAGE)

**Figure 4:**
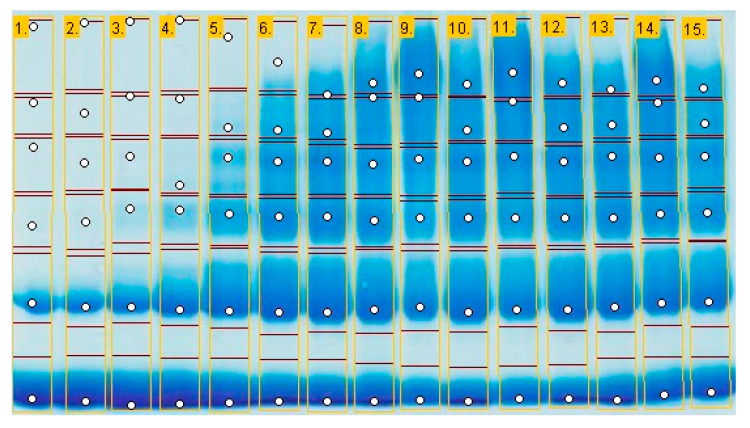
A 12% SDS-PAGE of 1.5 mM haemoglobin polymerization with varying concentrations of GA: 1; 2: 0 mM; 3: 0.5 mM; 4: 1 mM; 5: 3 mM; 6: 5 mM; 7: 7 mM; 8: 3 mM, room temperature (RT), week 8; 9: 5 mM, RT, week 8; 10: 3 mM, 40 °C, week 8; 11: 5 mM, 40 °C, week 8; 12: 3 mM, 1.2% isopropanol; 13: 3 mM, isopropanol, 60 °C, 5 h; 14: 5 mM, isopropanol, 60 °C, 5 h; 15: 3 mM isopropanol, 40 °C, week 8. For a similar experiment with myoglobin, see Figure S14. See also Figure S15 for monitoring the GA UV-vis spectra upon incubation at 60 °C.

Glutaraldehyde is extensively employed for the polycondensation of hemoglobin (Hb) and other proteins [20,21,22]. Here, Hb and myoglobin were employed in condensation reactions, under conditions similarly employed previously for other proteins [23]. Figure 4 shows such data, where the efficiency of the condensation was estimated using SDS-PAGE, with Table 3 showing the changes in relative proportions of fractions of various molecular weights. Lanes 8 and 10 show Hb polymerization with a 3 mM GA solution incubated for 8 weeks at room temperature and at 40 °C, respectively (to be compared with lane 5). Lanes 9 and 11 show the same experiments, now with 5 mM. Lanes 12–15 repeat the same experiments now for GA incubated in the presence of isopropanol, a typical ingredient in biocides, so as to assess its effect on GA stability; in this case, an accelerated protocol of aging was also employed, heating the GA samples (prior to reaction with Hb) to 60 °C for up to 24 h. One may expect that, upon incubation, the decrease in GA concentration would lead to decreases in the relative contributions of the higher molecular weight fractions. Yet, the incubated samples all featured stronger contributions of the higher molecular weight bands, compared to their reference non-incubated samples from the calibration set. For instance, the 3 mM sample incubated for 8 weeks had a relative intensity of 0.65 for the highest molecular weight region of the lane, compared to 0.00 for the reference non-incubated sample. In the case of the 5 mM sample, the highest molecular weight fraction even reached 1.00. On the other hand, the 3 mM sample incubated for 8 weeks at 40 °C expectedly showed slightly lower contributions of the higher molecular weight bands compared to the sample incubated at room temperature, which was likewise the case for the 5 mM sample at 8 weeks (40 °C vs. room temperature (RT)). 

The isopropanol-containing sample at 3 mM GA already showed a different molecular weight profile in the non-incubated sample vs. the non-isopropanol sample (lanes 5 vs. 12, respectively), suggesting that indeed isopropanol affects the crosslinking ability of GA. Incubation of the isopropanol-containing sample at 60 °C for 5 h drastically decreased the relative contributions of the higher molecular weight fractions; however, this trend was partially reversed when incubation was continued up to 24 h. Incubation for 8 weeks at 40 °C also decreased the contributions of the higher molecular weight bands and increased the relative contribution of the non-polymerized band.

These data did confirm that, over time, GA was gradually consumed from the solution. However, they also suggested that some of the GA derivatives formed during storage (dimers, oligomers, etc.; cf. Scheme 1) may have been more efficient crosslinkers than the monomer. This then put a limit on the degree to which an SDS-PAGE assay may be employed for assessing the concentration of GA solutions. 

### 2.6. UV-VIS Spectra

**Figure 5:**
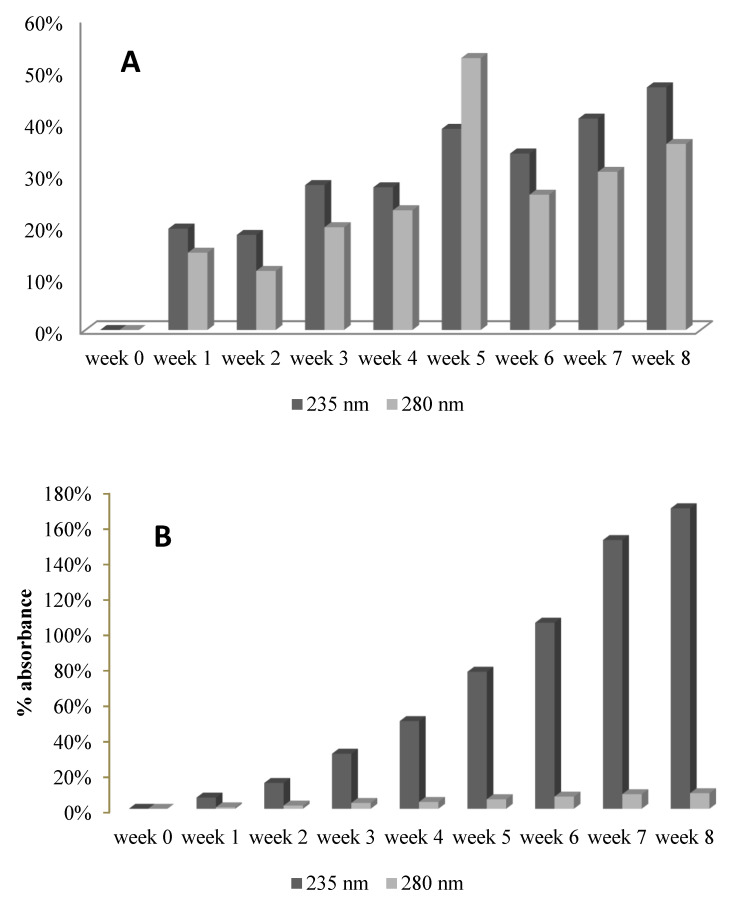
Evolution of absorbance in the UV-VIS spectra of 28.2 mM GA, at room temperature (**A**) and at 40 °C (**B**), for 8 weeks. See also Figures S10–S13.

Figure 5 shows the evolution of the UV-VIS spectrum of GA over time. Especially above room temperature (but also depending on other factors, such as pH or the presence of two other ingredients of biocides—isopropanol and a surfactant—see also the Supplementary Information), significant changes occur, with increases in the two maxima at 235 and at 280 nm. 

**Figure 6:**
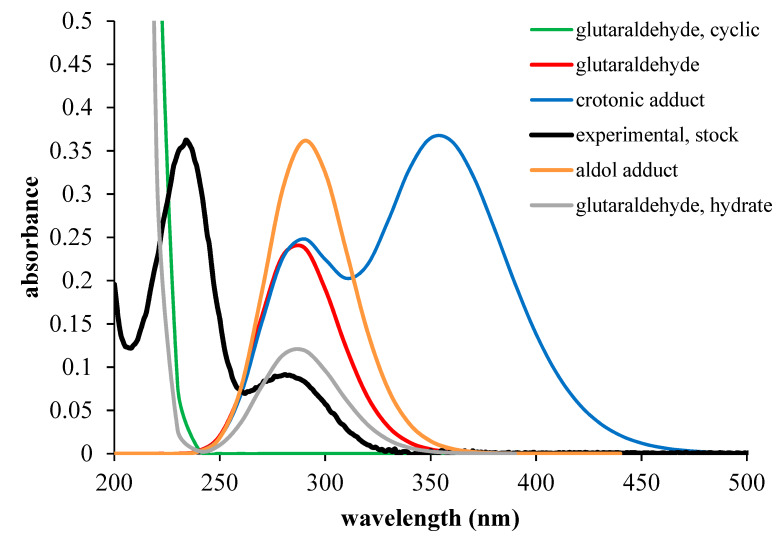
Comparison of the UV-VIS spectra of GA solutions, with spectra computed for GA and for a number of its reaction products.

The UV-VIS spectra of GA featured two major bands at 235 and 280 nm (Figure 5), previously assigned to sigma and/or pi transitions in the carbonyl groups. In order to clarify this issue, DFT calculations were employed for computing the UV-VIS spectrum of GA, as well as of some of its derivatives (cf. Figure 6). The computed spectrum of GA showed only a band at 290 nm, assigned to n-pi* transitions (orbitals shown in Table S2). This implied that the 235 nm band, already present in the commercial stocks of GA, was due to products of GA auto-condensation/degradation. As seen above (Figure 5), in the incubated samples, this band drastically increased in intensity over time, suggesting a distinct increase in the concentration of GA degradation products. Figure 6 shows DFT-computed spectra for a few of these products; on the basis of these, one may conclude that incubation of GA leads to relative increases in the contributions of less-conjugated chromophores such as hydrates or cyclic adducts, that is, those that display maxima below 250 nm rather than at 280 nm or higher (note that the time-dependent DFT (TD-DFT) protocol has an inherent error that may reach tens of nanometers [24]). 

## 3. Materials and Methods 

UV-VIS spectra were measured using a Varian Cary 50 spectrophotometer (Agilent, Inc., Santa Clara, CA, USA) or a Spark 10M microplate reader (Tecan GmbH, GrödigM Austria,). Raman spectra were acquired on a Renishaw inVia Raman Microscope (Renishaw, Wotton-under-Edge, United Kingdom) (using 50% of the 80 mW HeCd laser emitting at 442 nm, and recording with 10 s exposure time and 2 accumulations at room temperature. 

The NMR spectra were recorded at room temperature unless otherwise stated, after diluting the sample (at concentrations indicated in text and figure legends) with D_2_O (1:1 volume ratio), on a 400 MHz Bruker instrument (Bruker BioSpin GmbH, Rheinstetten, Germany). 

Chromatography experiments were performed using an Agilent 1210/6410 LC-MS/MS system (Agilent, Inc., Santa Clara, CA, USA). The Agilent LC1200 HPLC system was coupled to an Agilent 6410B triple quadrupole mass spectrometer. The HPLC column was a Phenomenex Kinetex F5 (reverse phase pentafluorophenyl), 50 × 2.1 mm, 2.6 μm particle size, 100 Å pore size (Phenomenex, Torrance, CA, USA). HPLC-grade acetonitrile and water, and LC/MS grade formic acid (Hypersolv Chromanorm) were purchased from VWR (VWR, Radnor, PA, USA). The mobile phase for liquid chromatography was 65% MeCN, 35% HPLC-grade water containing 0.1% HCOOH at a mobile phase flow rate of 0.3 mL/min, column temperature 35 °C, injection volume 5 μL, runtime 5 min. The mass spectrometer was used in ESI-positive ionisation mode. Spectra were recorded in MS2 Scan mode (TIC: total ion chromatograms), using a capillary voltage of 4 kV, source temperature 350 °C, collision energy 0 V, fragmentor voltage 90/100 V, scan range m/z 40–300 amu (see also the Supplementary Information). The samples were diluted with 2:1 MeCN/H_2_O prior to injection into HPLC, in order to have an appropriate concentration for LC/MS analysis.

Titration with glycine entailed monitoring the formation of imines at 270 nm. GA solutions used for titration were from Sigma-Aldrich (Merck KGaA, Darmstadt, Germany), grade II, 25%, in H_2_O. The experiments were performed in water and the samples were collected weekly for up to 8 weeks. Calibration curves were constructed using glycine in 10-fold excess over glutaraldehyde, at GA concentrations of 1.2, 1.8, 2.4, and 3.2 mM (cf. Supplementary Information). Under all explored conditions, the GA+glycine reaction was not instantaneous and not saturating (i.e., at up to glycine concentrations as high as 250 mM, the maximum absorbance of the GA–glycine adduct at fixed GA concentrations increased proportionally with the glycine concentration; a lower estimate of the extinction coefficient under these conditions was 230 M^−1^ cm^−1^). Therefore, two calibration curves were constructed, which gave identical results, as shown in Figure S9; one based on the initial rate of absorbance increase after mixing GA with glycine, and one based on the absorbance reached at 10 min after mixing GA with glycine at varying GA concentrations.

Redox reactivity was assessed using the Folin–Ciocalteu method, as previously described [25,26].

For SDS-PAGE analysis, polymerization of hemoglobin (Hb) and/or myoglobin (Mb) with GA was performed in Eppendorf tubes, each provided with a magnetic stirrer, in PBS (phosphate-buffered saline), following previously described procedures [20,21]. The reaction mixture was stirred for 2 h, then sodium borohydride was added for 30 min, and after, a Tris buffer solution was added to stop the reaction. The relative intensities of bands on each lane were computed using the Gel Analyzer 2010a software (gelanalyzer.com), as previously described [27] Calibration lanes (1–7 in Figure 4, spanning 0 to 7 mM GA with fresh stock solutions) were included for comparison with the GA samples to be studied.

DFT calculations (geometry optimizations, NMR calculations) were performed using the Gaussian09 software package (Gaussian, Wallingford, CT, USA); the structures were optimized using the B3LYP hybrid functional and 6–311+G** basis set [28]; the UV-VIS spectra were computed using the time-dependent (TDDFT) protocol (*N* = 10), as previously described [24]. The absence of imaginary frequencies was verified in order to ensure that proper minima were obtained.

## 4. Conclusions

The UV-VIS spectra of GA featured bands at 230–235 and 280 nm; the latter can be assigned to n-pi* transitions whereas the former can be assigned to degradation products (cf. DFT calculations). The UV-VIS spectrum changed significantly over time, especially at 40 °C, and mainly at 235 nm, with many possible condensation products. Raman spectra confirmed that, at higher temperatures, measurable changes occurred; a decrease in the relative ratios of the peaks at 1440 cm^−1^ (CH_2_ vibrations) vs. 1710 cm^−1^ (carbonyl vibrations) could be taken as evidence for the transformation of GA initiated by aldol-type condensation and hydrate formation. LC-MS data confirmed the instability of the GA solutions over time, whereas NMR additionally identified rapid temperature-dependent equilibria consistent with entropically disfavored reactions (condensation, hydration). Titration with glycine, followed at 270 nm, confirmed the spectral data; 2 weeks at 40 °C appeared to reduce the number of titratable carbonyl groups to a degree, equating 100% bi-molecular condensation of all GA in solution (i.e., where no free/intact/monomeric GA remained in solution). SDS-PAGE experiments revealed an unpredictable character of GA over time in the polymerization process. Protein crosslinking reactions suggested some of the degradation products of GA (dimer, oligomers) may have been more efficient than the monomer in protein crosslinking. Of the methods explored here for estimating the integrity of GA solutions, the most convenient appeared to be titration with glycine using the absorbance at 270 nm. The NMR spectrum, via the unique signal of the aldehyde proton at ≈9 ppm, also gave clear results, although this required more complicated instrumentation and was only feasible at relatively high GA concentrations. Notably, both of these methods assayed the free aldehyde groups, so that interpretation of the results had to take into account the GA byproducts that contained such functional groups. HPLC measurements had the disadvantage of processing the GA samples at high temperatures, which was expected to modify their chemical composition; as well as this, complete separation of products did not appear to be feasible. Insofar as testing the potential efficiency of the GA solutions as biocides, SDS-PAGE analysis of Hb polymerized with GA appeared to be the most relevant chemical test, showing that the GA byproducts could still act in this respect. Overall, these data reconfirmed the usefulness of reactivity-based vs. concentration-based assays for testing biocides, and suggested that the most appropriate tests for biocide conformity should still be the microbiological tests.

## Figures and Tables

**Table 1:** Thermodynamic parameters derived from the temperature dependence of the glutaraldehyde solution NMR spectra.

T (K)	ΔS (kcal/mol*K)	ΔH (kcal/mol)	ΔG (kcal/mol)
293	−0.02	−7.78	−1.27
323			−0.60
343			−0.15

**Table 2:** LC-MS chromatographic results and corresponding notable MS data (where assignments could be made). Chromatograms and spectra are shown in the Supplementary Information Figures S5–S8.

Sample	Peak 1 (Rf/rel. area)	Peak 2 (Rf/rel. area)	Notable MS Signals in Peak 1	Notable MS Signals in Peak 2
GA control, 0.3 M	0.573/100	0.855/41	101 (GA), 119 (II), 165 (VI/X minus water), 183 (VI/X, or IX minus water)	N.D.^a^
GA 0.3 M incubated 4 weeks at 40 °C	0.632/5	0.829/100	N.D.^a^	201 (IX), N.D.^a^
GA control, 2.5 M	0.581/100	0.837/20	101 (GA), 165 (VI/X minus water), 183 (VI/X, or IX minus water)	101 (GA), 119 (II), 183 (VI/X), or IX minus water), N.D.^a^
GA 2.5 M, incubated for 5 h at 70 °C	0.581/100	0.837/18	101 (GA), 165 (VI/X minus water), 183 (VI/X, or IX minus water)	101 (GA), 183 (VI/X, or IX minus water), N.D.^a^

^a^ Most or all of the signals were not assignable to any of the structures of Scheme 1.

**Table 3:** Profile of relative intensities of protein bands.

Sample / ≈MW (kDA)	250	130	100	70	55	35
0 mM GA	0	0	0	0.01	0.49	1
0.5 mM GA	0.01	0	0.01	0.06	0.61	1
1 mM GA	0	0	0.03	0.18	0.81	1
3 mM GA	0	0.05	0.34	0.49	1	0.82
5 mM GA	0.08	0.43	0.65	0.56	1	0.67
7 mM GA	0.28	0.51	0.68	0.58	1	0.67
3 mM GA, RT, week 8	0.65	0.64	0.58	0.57	1	0.77
5 mM GA, RT, week 8	1	0.6	0.52	0.36	0.75	0.52

## Supplementary Materials

Supplementary materials can be found at https://www.mdpi.com/1422-0067/21/9/3372/s1.

## Abbreviations

MDPI	Multidisciplinary Digital Publishing Institute
DOAJ	Directory of open access journals
TLA	Three letter acronym
LD	linear dichroism

## References

[1] Ballantyne B., Jordan S.L. (2001). Toxicological, medical and industrial hygiene aspects of glutaraldehyde with particular reference to its biocidal use in cold sterilization procedures. J. Appl. Toxicol..

[2] Power E.G.M., Russell A.D. (1988). Assessment of “Cold Sterilog Glutaraldehyde Monitor”. J. Hosp. Infect..

[3] Gannon P.F., Bright P., Campbell M., O’Hickey S.P., Burge P.S. (2008). Occupational asthma due to glutaraldehyde and formaldehyde in endoscopy and x ray departments. Thorax.

[4] Jolibois B., Guerbet M., Vassal S. (2002). Glutaraldehyde in hospital wastewater. Arch. Environ. Contam. Toxicol..

[5] Barnes A.R. (1993). Determination of glutaraldehyde in solution as its bis-2,4-dinitrophenylhydrazone derivative; determination of geometrical isomer ratios. Pharm. Acta Helv..

[6] Pieraccini G., Bartolucci G., Pacenti M., Dugheri S., Boccalon P., Focardi L. (2002). Gas chromatographic determination of glutaraldehyde in the pentafluorobenzyl hydroxylamine on a solid-phase microextraction fibre. J. Chromatogr. A.

[7] Kang H.I., Shin H.S. (2016). Determination of glutaraldehyde in water samples by headspace solid-phase microextraction and gas chromatography-mass spectrometry after derivatization with 2,2,2-trifluoroethylhydrazine. J. Chromatogr. A.

[8] Kang H.I., Shin H.S. (2016). Sensitive determination of glutaraldehyde in environmental water by derivatization and gas chromatography-mass spectrometry. Anal. Methods.

[9] Pranaityte B., Padarauskas A., Dikčius A., Ragauskas R. (2004). Rapid capillary electrophoretic determination of glutaraldehyde in photographic developers using a cationic polymer coating. Anal. Chim. Acta.

[10] Shaw J., Frigerio A. (1969). A Simple Method for Determination of Glutaraldehyde. J. Histochem. Citochemistry.

[11] Isabelle M., Catherine D., Bertrand Michel J., Waldron Karen C. (2004). Glutaraldehyde: Behavior in aqueous solution, reaction with proteins, and application to enzyme crosslinking. Biotechniques.

[12] Scurtu F., Zolog O., Iacob B., Silaghi-Dumitrescu R. (2014). Hemoglobin-albumin cross-linking with disuccinimidyl suberate (DSS) and/or glutaraldehyde for blood substitutes. Artif. Cells Nanomedicine Biotechnol..

[13] (2016). Hospital Scandal Brings Down Romanian Pharma Co.. Forbes.

[14] (2016). Romanian prosecutors make first arrest in the diluted disinfectants scandal | Romania Insider. Romania-Insider.

[15] Bowes J.H., Cater C.W. (1966). The reaction of glutaraldehyde with proteins and other biological materials. J. R. Microsc. Soc..

[16] Guthrie J.P. (1978). Equilibrium constants for a series of simple aldol condensations, and linear free energy relations with other carbonyl addition reactions. Can. J. Chem..

[17] Kurz J.L. (1967). The Hydration of Acetaldehyde. I. Equilibrium Thermodynamic Parameters. J. Am. Chem. Soc..

[18] Okuda K., Urabe I., Yamada Y., Okada H. (1991). Reaction of glutaraldehyde with amino and thiol compounds. J. Ferment. Bioeng..

[19] Korn A.H., Feairheller S.H., Filachoine E.M. (1972). Glutaraldehyde: Nature of the reagent. J. Mol. Biol..

[20] Hathazi D., Mot A.C., Vaida A., Scurtu F., Lupan I., Fischer-Fodor E., Damian G., Kurtz D.M., Silaghi-Dumitescu R. (2014). Oxidative protection of hemoglobin and hemerythrin by cross-linking with a nonheme iron peroxidase: Potentially improved oxygen carriers for use in blood substitutes. Biomacromolecules.

[21] Arkosi M., Scurtu F., Vulpoi A., Silaghi-Dumitrescu R., Kurtz D.M. (2017). Copolymerization of Recombinant P. gouldii Hemerythrin with Human Serum Albumin for Use in Blood Substitutes. Artif. Cells Blood Substitutes Biotechnol..

[22] Silva C.J.S.M., Sousa F., Gübitz G., Cavaco-Paulo A. (2004). Chemical Modifications on Proteins Using Glutaraldehyde. Food Technol. Biotechnol..

[23] Alayash A.I., Summers A.G., Wood F., Jia Y. (2001). Effects of glutaraldehyde polymerization on oxygen transport and redox properties of bovine hemoglobin. Arch Biochem Biophys..

[24] Attia A.A.A., Cioloboc D., Lupan A., Silaghi-Dumitrescu R. (2016). Multiconfigurational and DFT analyses of the electromeric formulation and UV–vis absorption spectra of the superoxide adduct of ferrous superoxide reductase. J. Inorg. Biochem..

[25] Everette J.D., Bryant Q.M., Green A.M., Abbey Y.A., Wangila G.W., Walker R.B. (2010). Thorough study of reactivity of various compound classes toward the folin-Ciocalteu reagent. J. Agric. Food Chem..

[26] Mot A.C.C., Bischin C., Muresan B., Parvu M., Damian G., Vlase L., Silaghi-Dumitrescu R. (2016). Antioxidant activity evaluation by physiologically relevant assays based on haemoglobin peroxidase activity and cytochrome c-induced oxidation of liposomes. Nat. Prod. Res..

[27] Silaghi-Dumitrescu R., Tomoiaga N., Jurco E. (2018). Variability in biochemical composition of milk among three representative breeds of dairy cows from Romania. Stud. Univ. Babes-Bolyai Chem..

[28] Frisch M.J., Trucks G.W., Schlegel H.B., Scuseria G.E., Robb M.A., Cheeseman J.R., Scalmani G., Barone V., Mennucci B., Petersson G.A. (2009). Gaussian 09, Gaussian 09 r. A1.

