# Supplementary material for: Stability of Glutaraldehyde in Biocide Compositions
Source: Int J Mol Sci. 2020 May 10;21(9):3372. doi: 10.3390/ijms21093372 (PMC7246990; doi:10.3390/ijms21093372)
Supplement: Supplementary file 1 [file ijms-21-03372-s001.pdf]

Supporting Information

On the stability of glutaraldehyde in biocide compositions

Alina Matei[1], Cristina Puscas[1], Iulia Patrascu[1], Maria Lehene[1], Julia Ziebro[2], Florina Scurtu[1], Monica Baia[3], Dan Porumb[1], Robert Totos[1], Radu Silaghi-Dumitrescu[1(⌧)]

1. *Department of Chemistry, Babes-Bolyai University, 1 Mihail Kogalniceanu street, Cluj-Napoca 400084, Romania*
2. *Department of Chemistry, Northern Kentucky University, Southgate, KY USA*
3. *Department of Physics, Babes-Bolyai University, 1 Mihail Kogalniceanu street, Cluj-Napoca 400084, Romania*

(⌧) *corresponding author: rsilaghi@chem.ubbcluj.ro*

As shown in Supporting Information Figure S1, glutaraldehyde solutions from varying sources/vendors display very similar Raman spectra. On the other hand, Figure S1 also reveals that simply diluting a 25% solution (~2.5 M) to 1 M has a distinct effect on the relative intensities of peaks in the 1600 cm^{-1} region (water stretching frequency) as well as in the most notable bands specific to glutaraldehyde (801, 1444 and 1716 cm^{-1}). This may be linked with the equilibria inherent to glutaraldehyde in water solutions – which are bound to be concentration-dependent.

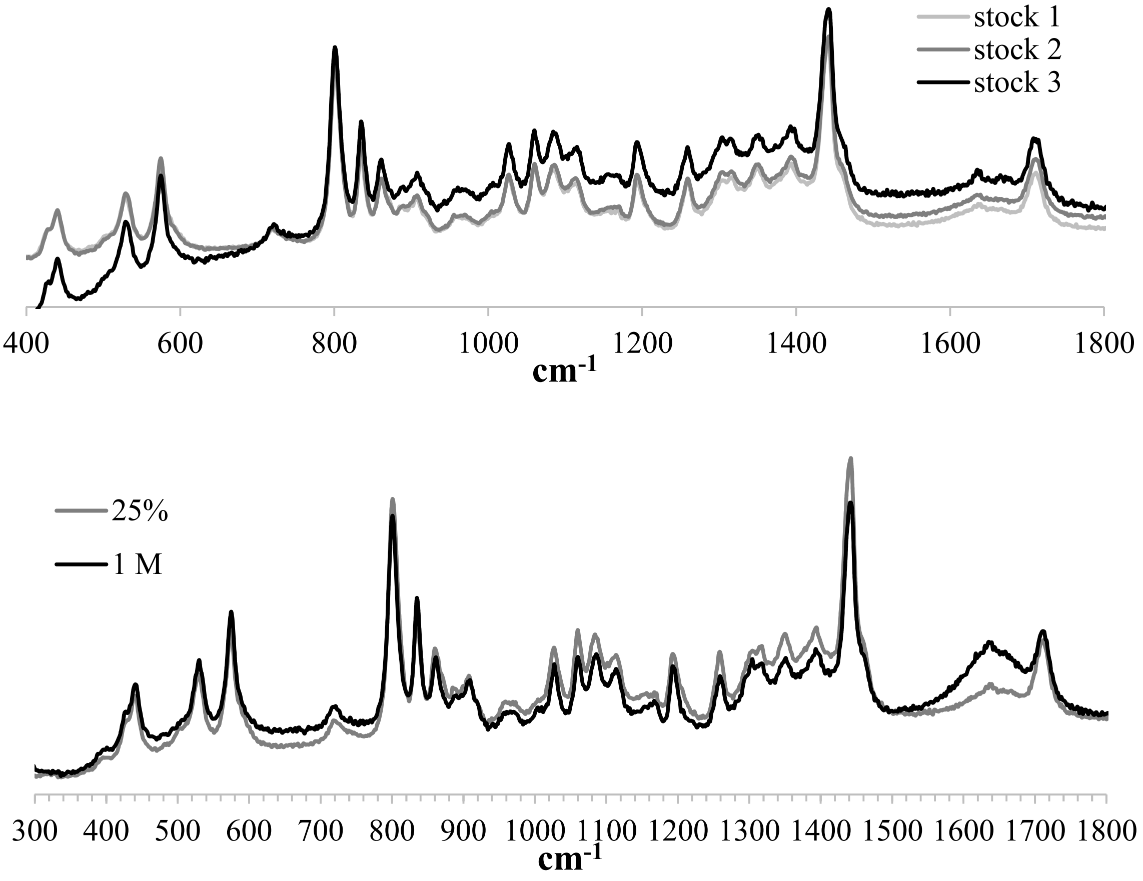

Figure S1. Top panel: Raman spectra of 1M glutaraldehyde. Stock 1-glutaraldehyde solution 25% Sigma-Aldrich 30.06.2016, stock 2- glutaraldehyde solution 25% Alfa-Aesar 12.09.2018, stock 3- glutaraldehyde solution 25% Sigma-Aldrich. Bottom panel: Raman spectra of 25% and 1 M glutaraldehyde. The 1 M spectrum is normalized to the 25% spectrum.

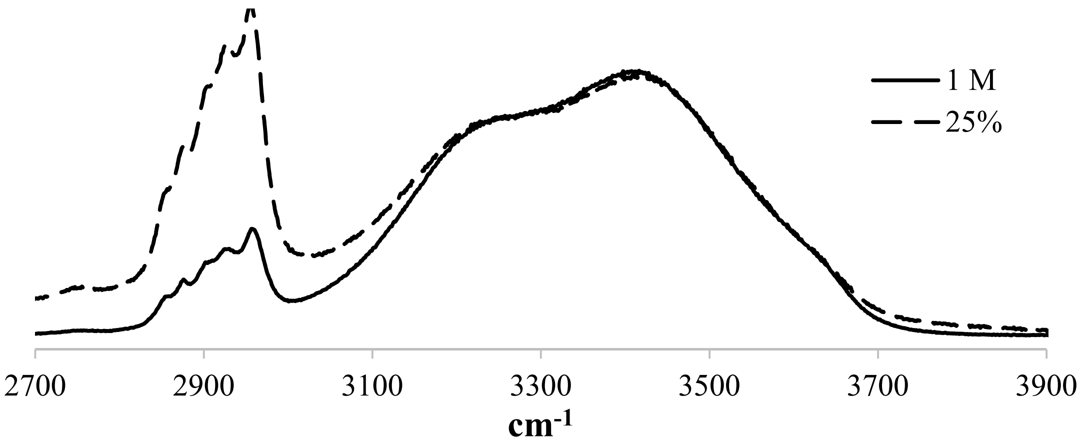

Figure S2. Raman spectra of glutaraldehyde at two different concentrations – 25% (2.82 M) and 1 M. Conditions as in Figure S1.

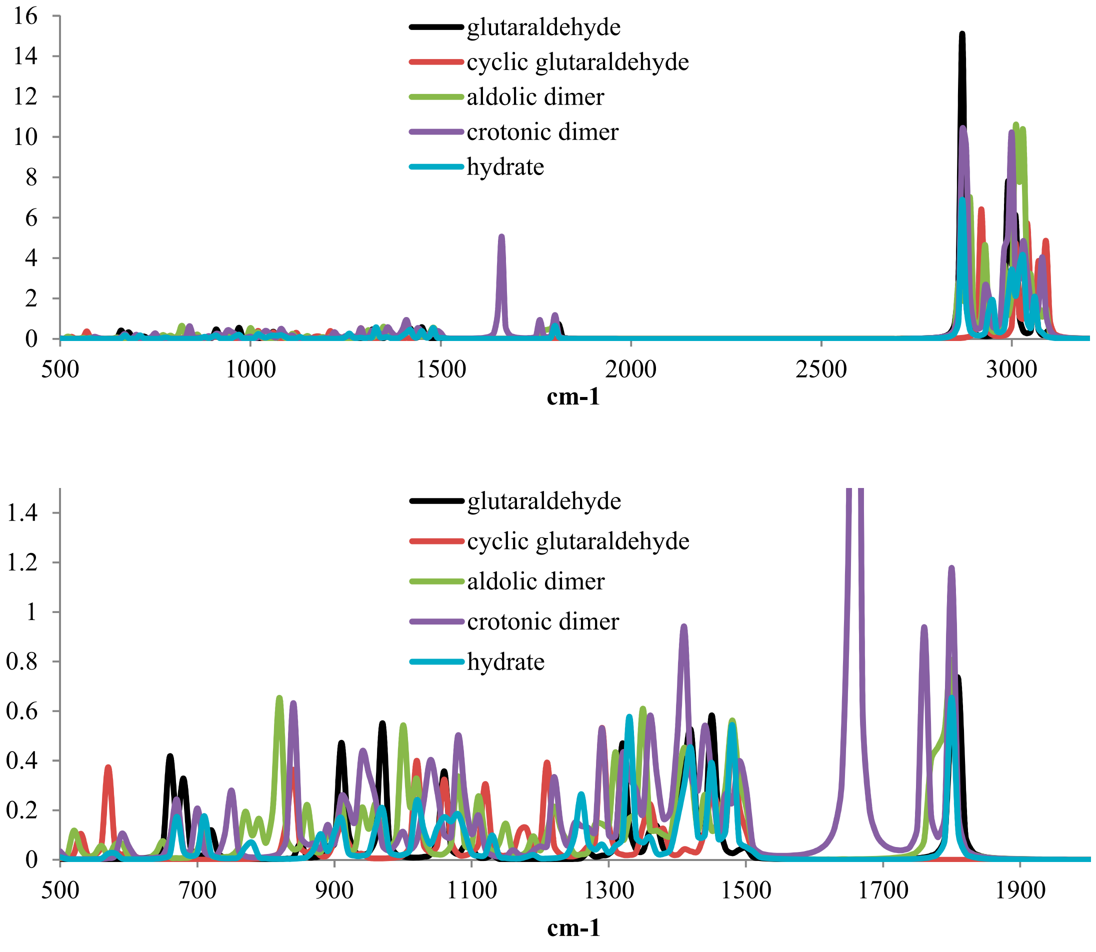

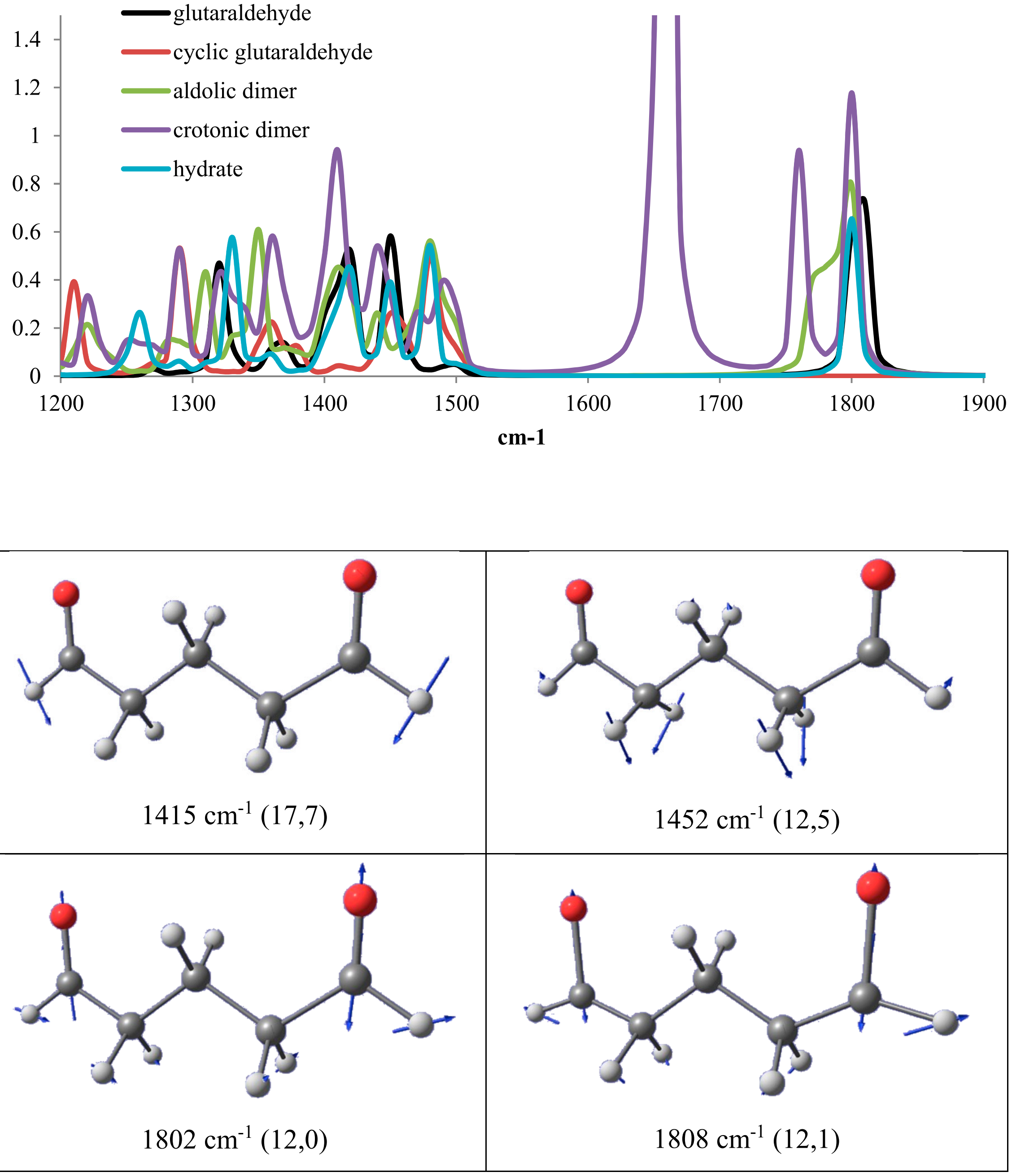

Figure S3. Raman spectra of glutaraldehyde and its derivatives, and representations of the molecular vibrations corresponding to the two most intense regions in the 1000-2000 cm^{-1} for glutaraldehyde, as computed at the B3LYP/6-311+G** level of theory.

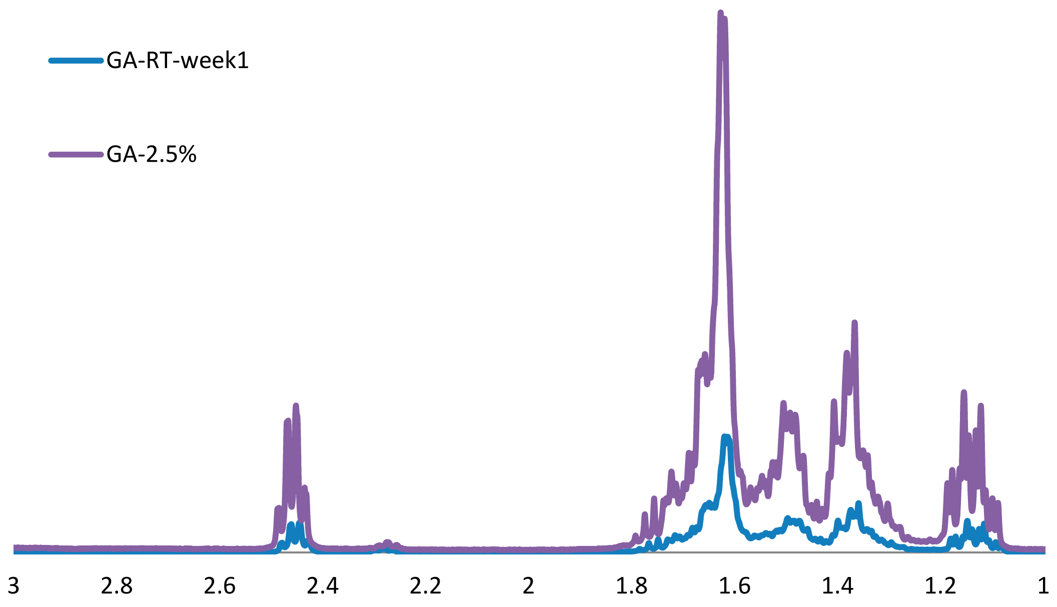

Figure S4. NMR spectra of GA before and after incubation for a week at room temperature.

Table S1. NMR signals (ppm) for the glutaraldehyde and other products computed at the B3LYP/6-311+G** level of theory.

Proton type	GA	Cyclic GA	GA hydrate	Aldolic dimer	Crotonic dimer
Aldehyde region	10.2		10.2	10.3, 10.1	10.3, 10.2, 10.1
Unsaturated region		4.7	5.1	4.0	6.0
Highest at aliphatic region	2.2	1.7	2.4, 2.3	2.6, 2.4	3.5, 2.8, 2.7, 2.5, 2.4
Rest of aliphatic region	1.8	1.5, 1.4, 1.3	1.9, 1.6, 1.5, 1.5, 1.4, 1.3	1.8, 1.7, 1.6, 1.3, 1.2, 0.7	2.0, 1.8, 1.5, 1.4

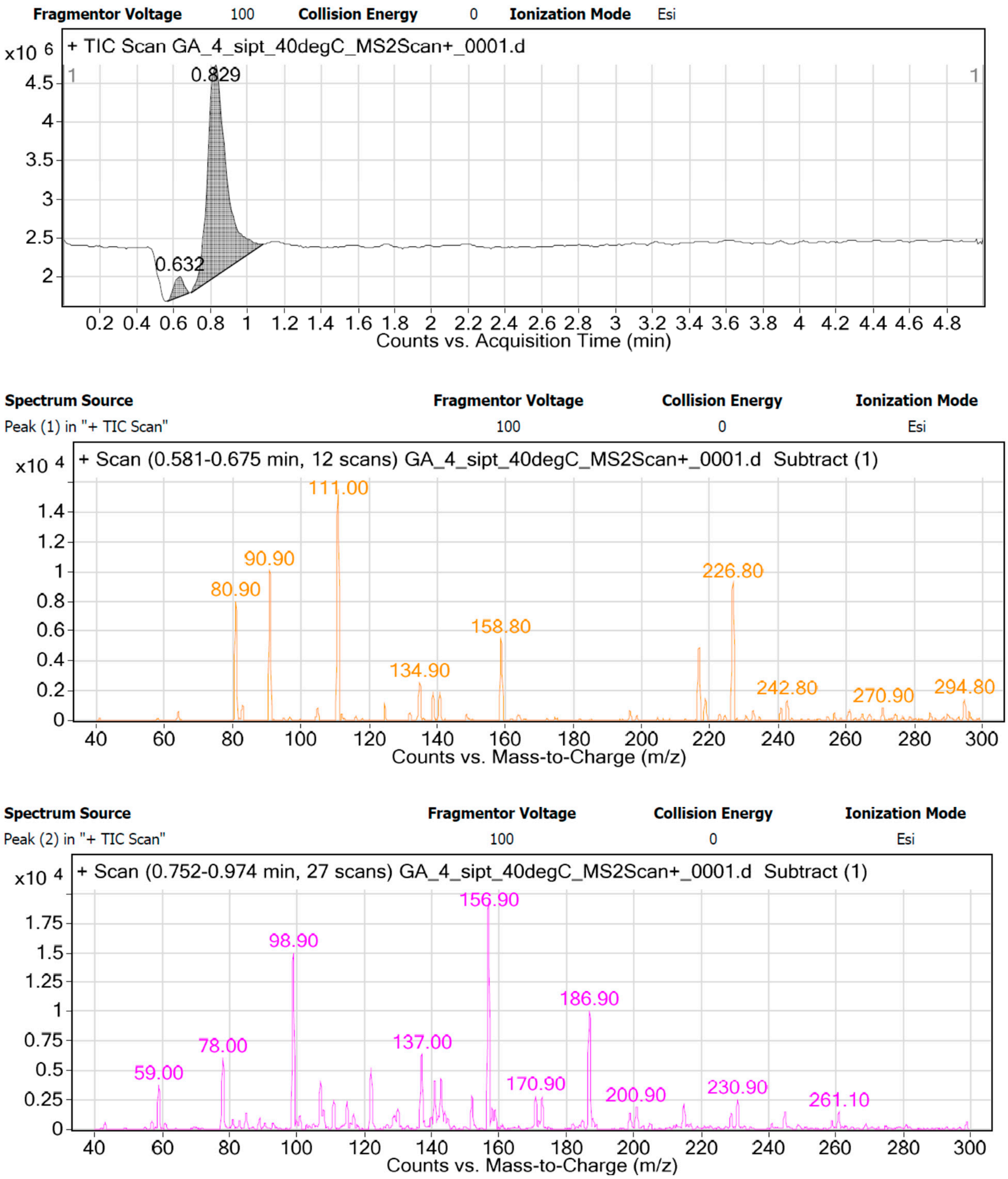

Figure S5. LC-MS data for a GA sample incubated 4 weeks at 40°C (at 0.3 M GL).

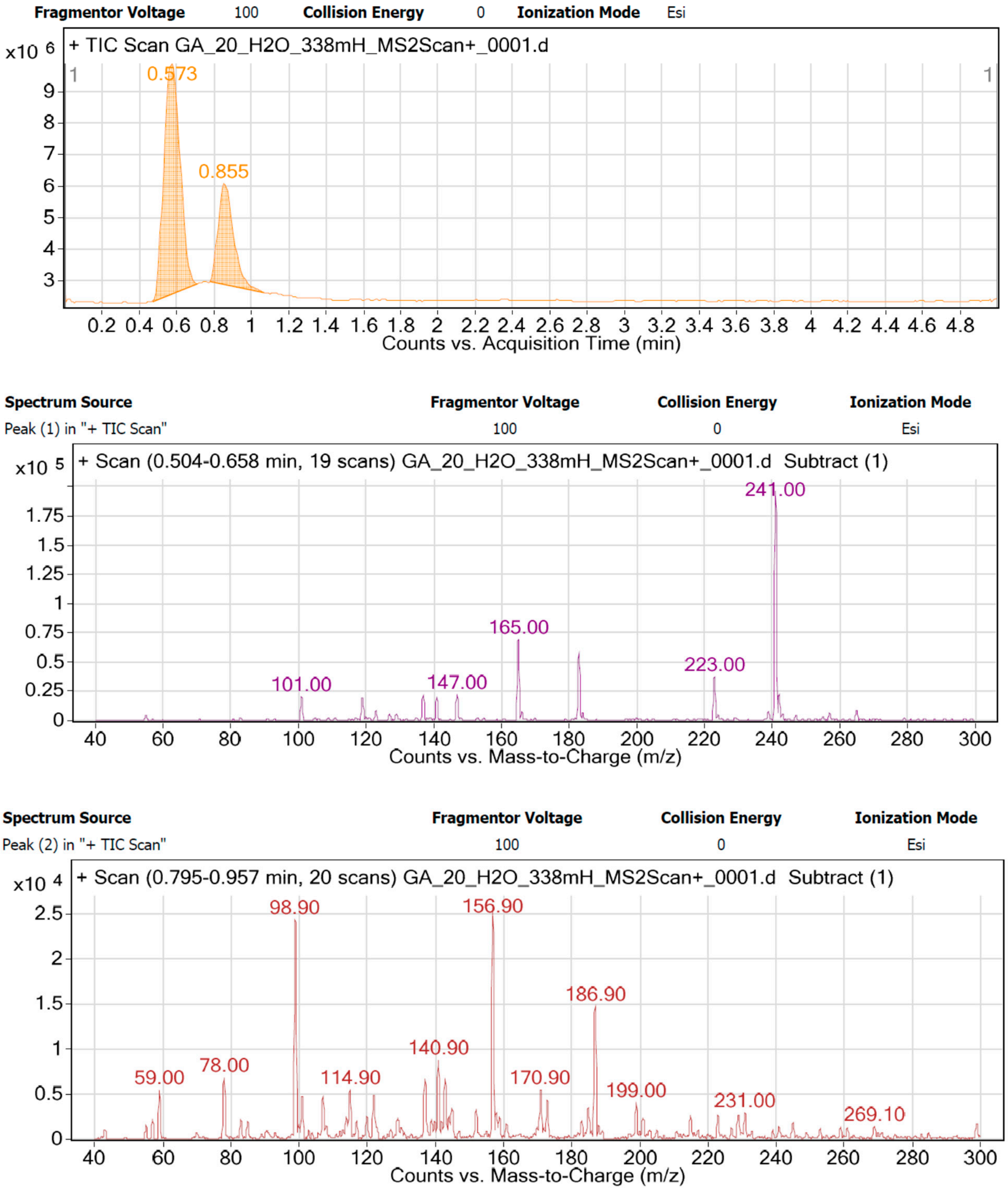

Figure S6. LC-MS data for the control sample before incubation for 4 weeks at 40°C (at 0.3 M GA).

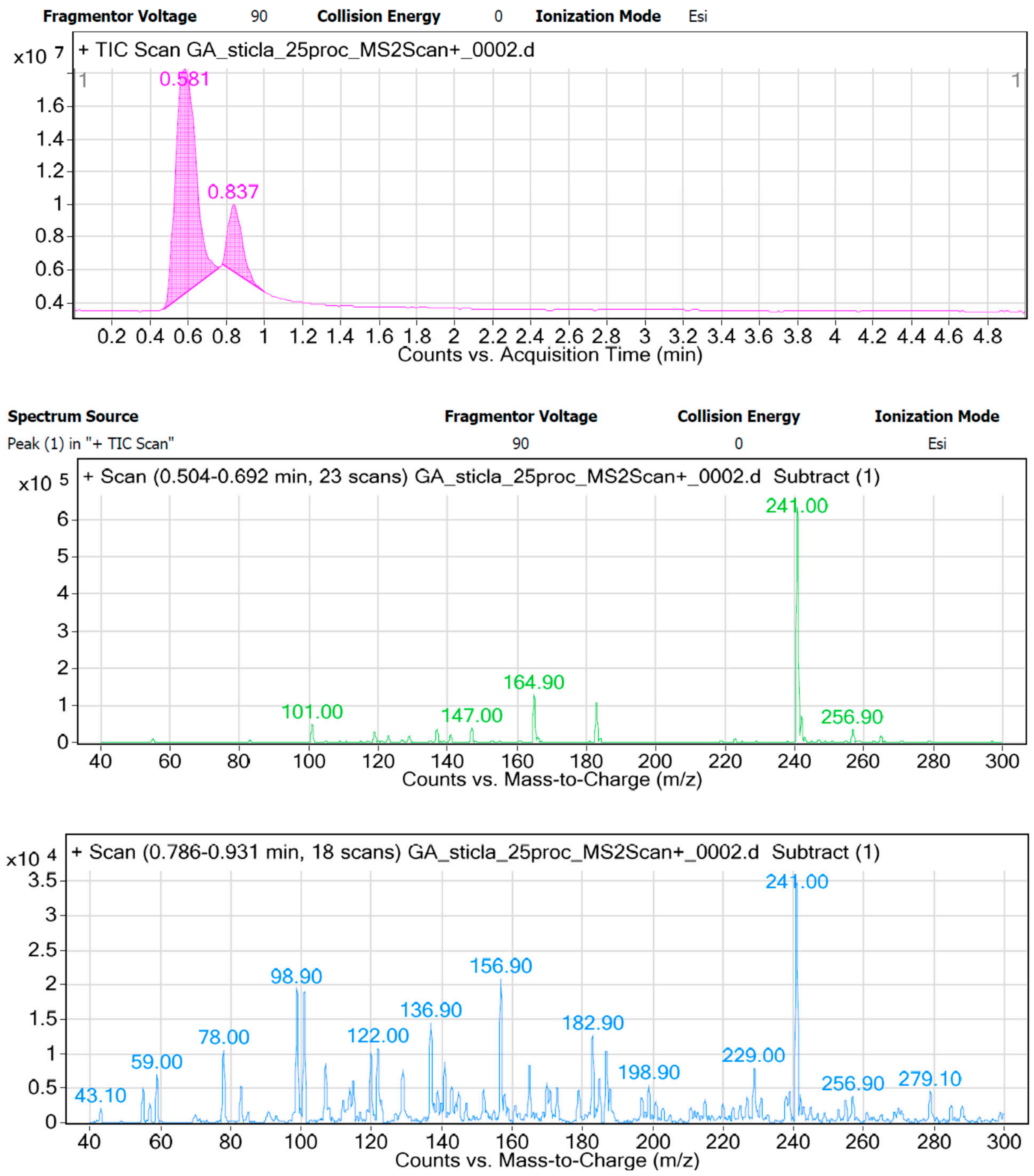

Figure S7. LC-MS data for the 25% (2.5 M) GA stock solution.

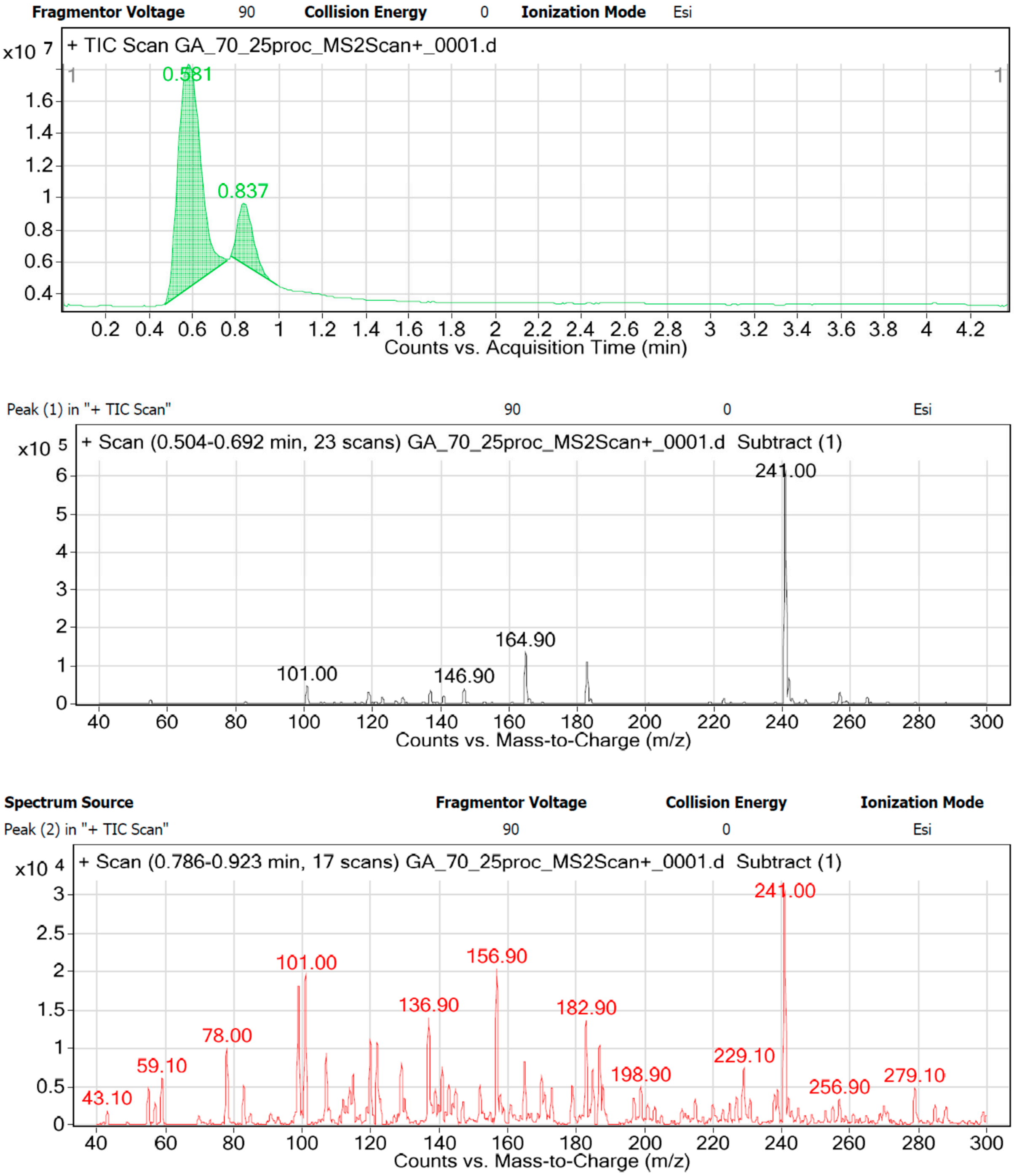

Figure S8. LC-MS data for the 25% (2.5 M) GA stock solution after incubation at 70°C for 5 hours.

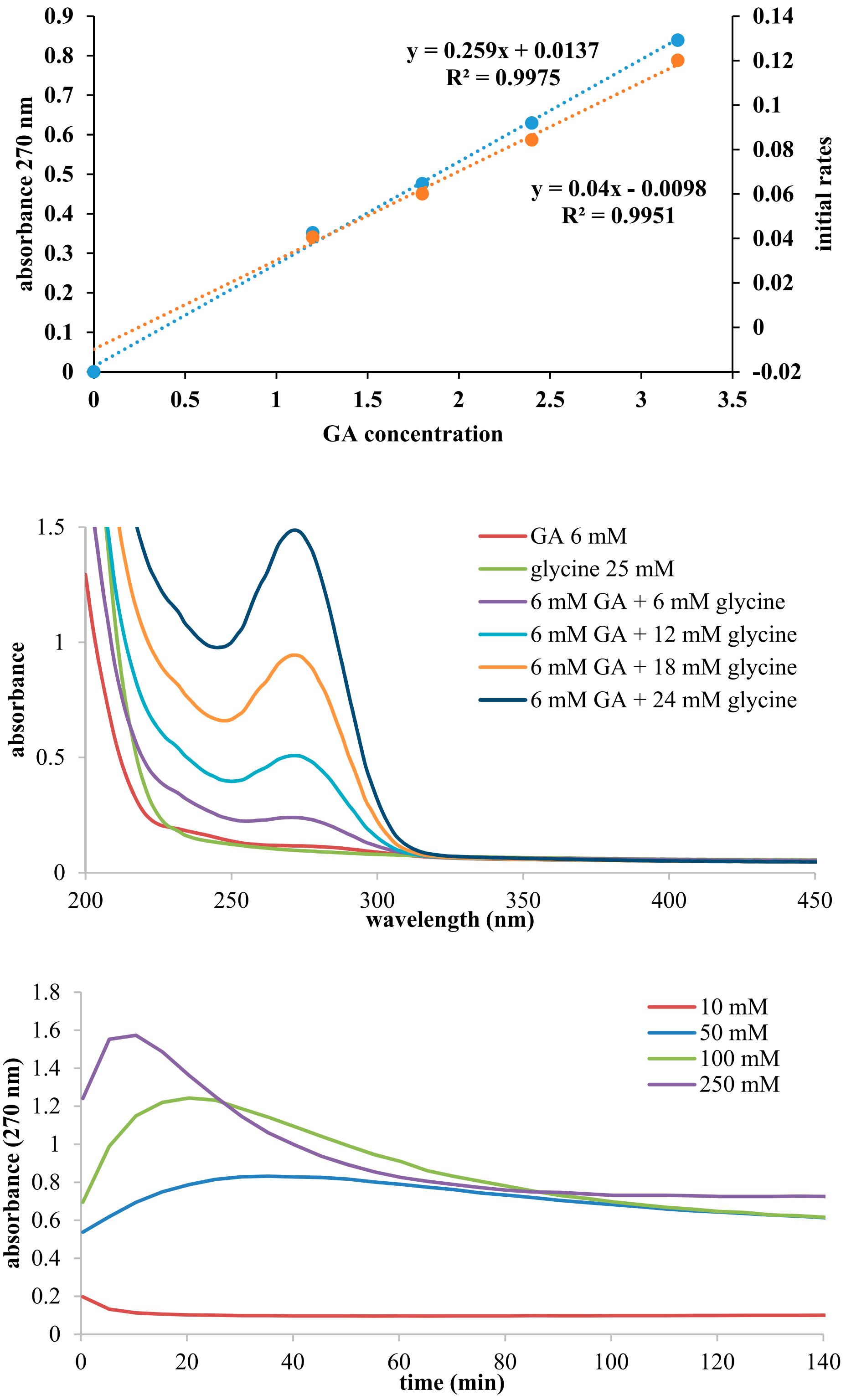

Figure S9. Top panel calibration curve at 270 nm, using 32 mM glycine and varying concentrations of glutaraldehyde (1.2-3.2 mM). Middle panel: UV-vis spectra of glycine, GA

and glycine-GA mixtures at indicated concentrations. Bottom panel: evolution of absorption at 270 nm for three different GA concentrations (indicated in Figure). All spectra are collected in water, at room temperature.

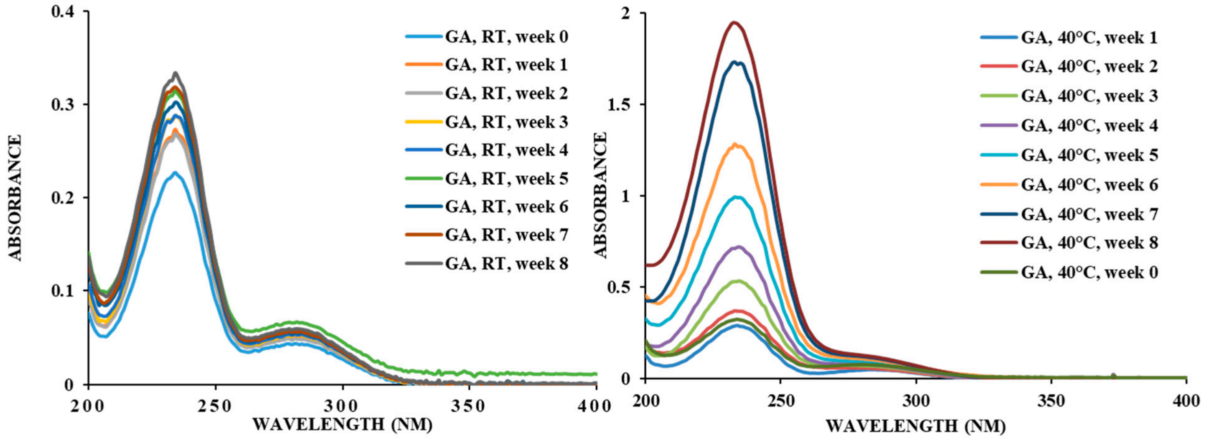

Figure S10. UV-vis spectra of 28.2 mM glutaraldehyde, at room temperature and 40°C, for 8 weeks.

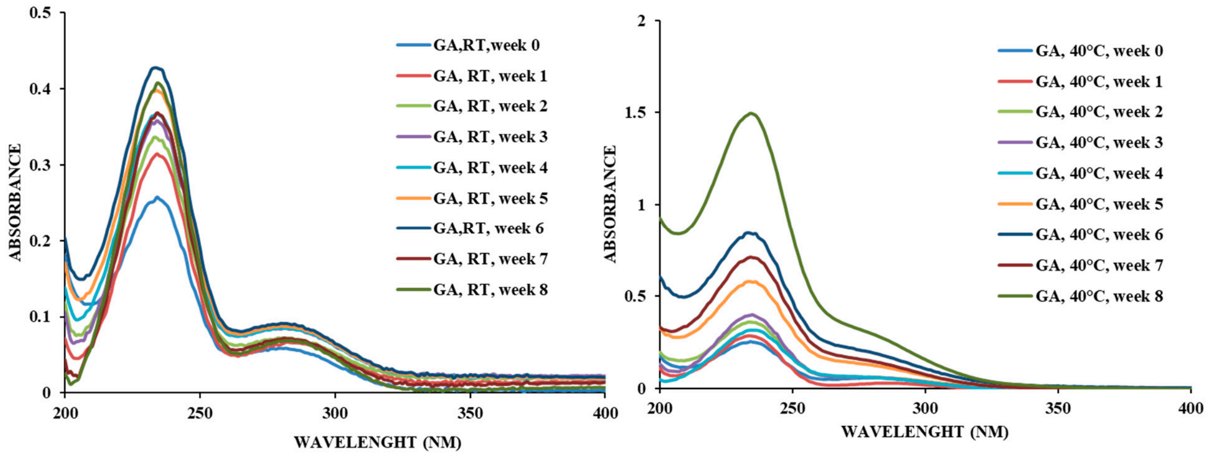

Figure S11. UV-vis spectra of 28.2 mM glutaraldehyde, 1.2% isopropanol, at room temperature and 40°C, for 8 weeks.

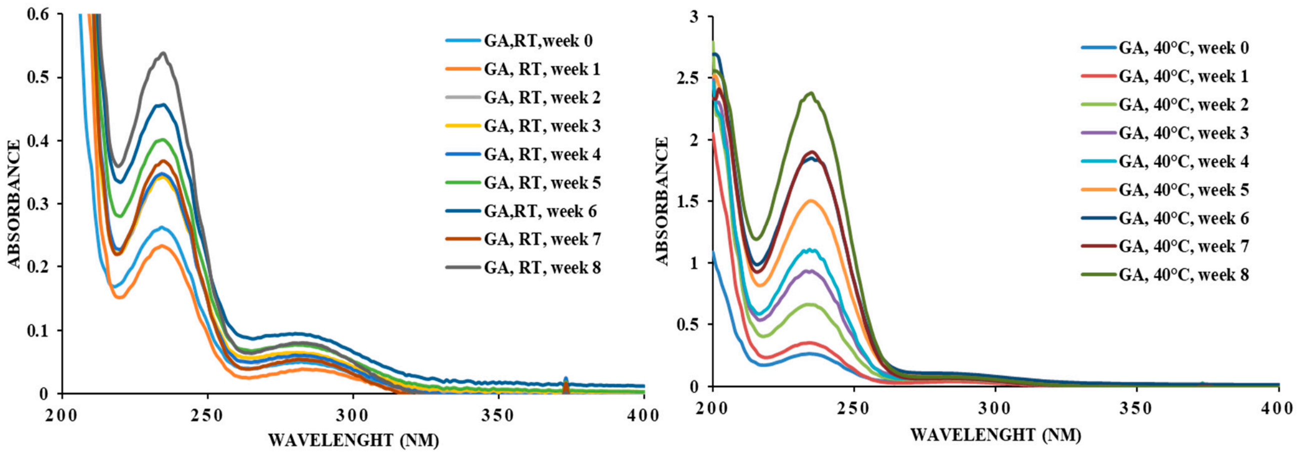

Figure S12. UvV-vis spectra of 28.2 mM glutaraldehyde, 1% n-cetyl-trimethylammonium bromide, at room temperature and 40°C, for 8 weeks.

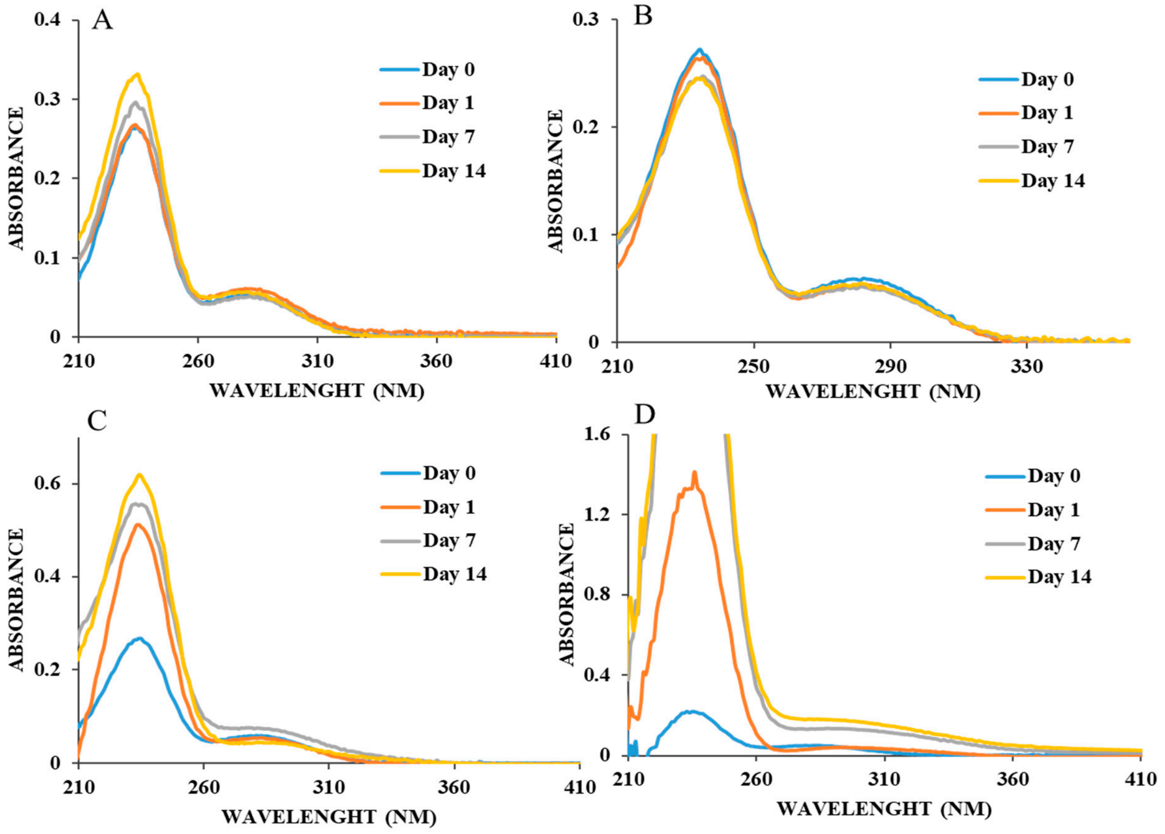

Figure S13. UV-vis spectra of 10 mM glutaraldehyde, in time, A) at pH 2 (100 mM monosodium phosphate), B) water, C) at pH 7 (100 mM disodium phosphate) and D) at pH 11 (100 mM trisodium phosphate).

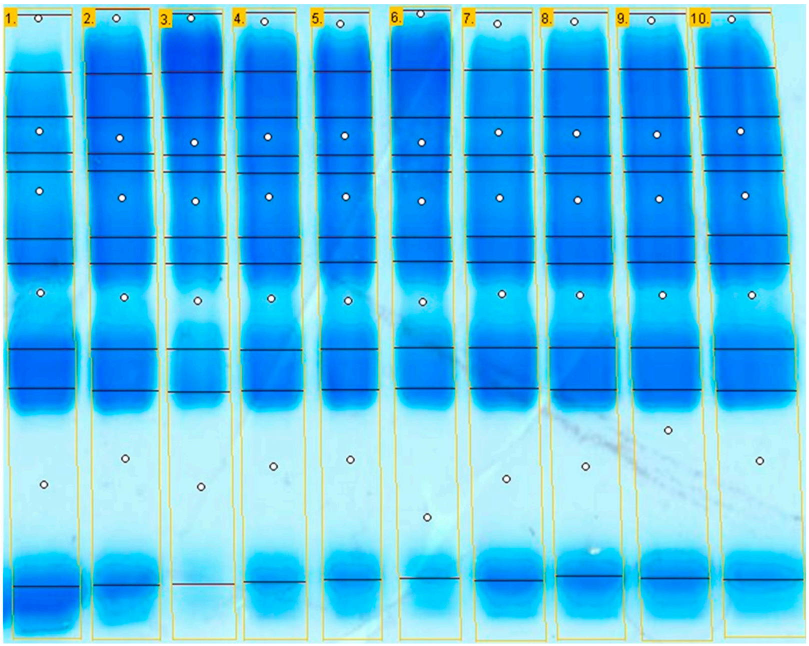

Figure S14. 12% SDS-PAGE of 1.5 mM myoglobin polymerization with varying concentrations of glutaraldehyde: 1: 5 mM, 2: 8 mM, 3: 10 mM, 4: 8 mM, 5h, 5: 8 mM, 20h, 6: 8 mM, 24h, 7: 8 mM, 1,2% isopropanol, t0, 8: 8 mM, isopropanol, 5h, 9: 8 mM, isopropanol, 20h, 10: 8 mM, isopropanol, 24h.

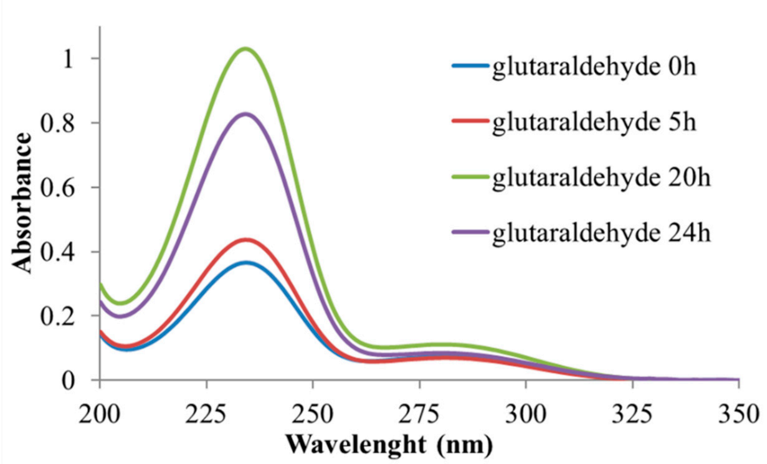

Figure S15. Evolution of absorbance in the UV-vis spectra of GA, at 60°C, for 24h.

Table S2. The four main orbitals contributing to the lowest energy transition in the UV-vis spectrum: 4.33 eV, 0.0004 oscillator strength, as computed at the B3LYP/6-311G++ level of theory. The HOMO-1->LUMO and HOMO->LUMO+1 excitations have essentially equal contributions to the 290 nm band.

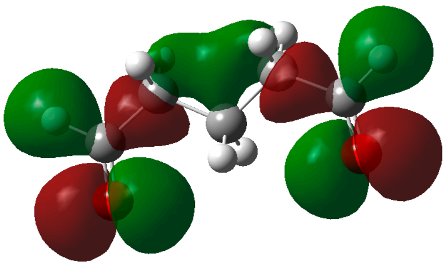 HOMO-1 (-7.53 eV)	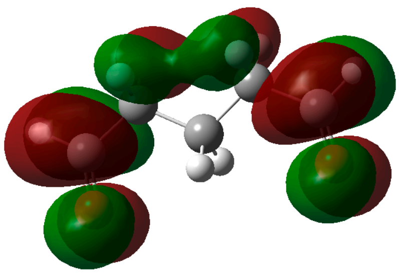 LUMO (-1.24 eV)
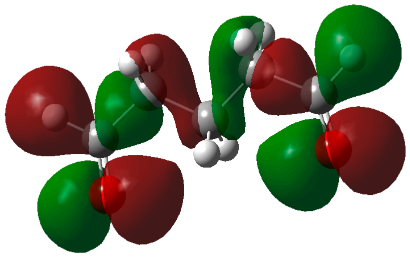 HOMO (-7.26 eV)	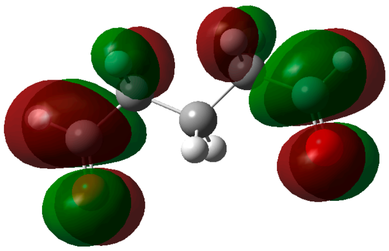 LUMO+1 (-1.04 eV)

Redox reactivity

Redox reactivity of the GA solutions was measured using the Folin-Ciocalteu method, in view of the fact that the aldehyde groups, as well as some of the functional groups in the degradation products, may display such reactivity. Sample of 10 mM glutaraldehyde from t0 and after 7 and 8 weeks incubated at 40°C were analyzed. The redox reactivity was determined using the equation obtained from the calibration curve of gallic acid (R^2=0.998). The resulting data, showing 16.24 ± 0.52, 22.02 ± 0.80, and 26.38 ± 0.62 µg GAE/mg GA for the samples at t0, 7 weeks and 8 weeks respectively, reveal a weak increase in redox reactivity. As such, they offer no direct measure of the GA concentration – but only one further confirmation of the change in the chemical composition of the GA solution over time.

Full Gaussian citation: Gaussian 09, Revision E.01, M. J. Frisch, G. W. Trucks, H. B. Schlegel, G. E. Scuseria, M. A. Robb, J. R. Cheeseman, G. Scalmani, V. Barone, B. Mennucci, G. A. Petersson, H. Nakatsuji, M. Caricato, X. Li, H. P. Hratchian, A. F. Izmaylov, J. Bloino, G. Zheng, J. L. Sonnenberg, M. Hada, M. Ehara, K. Toyota, R. Fukuda, J. Hasegawa, M. Ishida, T. Nakajima, Y. Honda, O. Kitao, H. Nakai, T. Vreven, J. A. Montgomery, Jr., J. E. Peralta, F. Ogliaro, M. Bearpark, J. J. Heyd, E. Brothers, K. N. Kudin, V. N. Staroverov, T. Keith, R. Kobayashi, J. Normand, K. Raghavachari, A. Rendell, J. C. Burant, S. S. Iyengar, J. Tomasi, M. Cossi, N. Rega, J. M. Millam, M. Klene, J. E. Knox, J. B. Cross, V. Bakken, C. Adamo, J. Jaramillo, R. Gomperts, R. E. Stratmann, O. Yazyev, A. J. Austin, R. Cammi, C. Pomelli, J. W. Ochterski, R. L. Martin, K. Morokuma, V. G. Zakrzewski, G. A. Voth, P. Salvador, J. J. Dannenberg, S. Dapprich, A. D. Daniels, O. Farkas, J. B. Foresman, J. V. Ortiz, J. Cioslowski, and D. J. Fox, Gaussian, Inc., Wallingford CT, 2013.
